# Transcriptomic analysis reveals pathogenicity mechanisms of *Phytophthora capsici* in black pepper

**DOI:** 10.3389/fmicb.2024.1418816

**Published:** 2024-11-18

**Authors:** Saranya Vijayakumar, Gayathri G. Saraswathy, Manjula Sakuntala

**Affiliations:** ^1^Plant Disease Biology Lab, Rajiv Gandhi Centre for Biotechnology, Thiruvananthapuram, India; ^2^Research Centre, University of Kerala, Thiruvananthapuram, India

**Keywords:** *Phytophthora*, black pepper, transcriptome, pathogenicity, quick wilt, effectors

## Abstract

The devastating disease “quick wilt” or “foot rot” is caused by the oomycete *Phytophthora capsici* Leonian and is affecting the economically significant spice crop black pepper (*Piper nigrum* L.). The details on the mechanism of interaction of *P. capsici* with its host black pepper remain poorly understood, hindering efforts to enhance disease resistance. To address this knowledge gap, we conducted an RNA-seq analysis to investigate the gene expression profile of *P. capsici* infecting black pepper. Comparative transcriptome analysis between axenic culture, and early and late infection stages of *P. capsici* revealed a substantial number of differentially expressed genes. Our findings demonstrate the induction of metabolic pathways, signaling cascades, and crucial pathogenicity-related processes during infection of black pepper by *P. capsici*. Specifically, we observed orchestrated expression of cell wall-degrading enzymes, effectors, and, detoxifying transporters at different infection time points, implicating their roles in pathogenicity. The expression patterns of key pathogenicity-associated genes, including effectors, were validated using reverse transcription quantitative real-time PCR. The effectiveness of agroinfiltration-mediated transient expression in black pepper for functional studies of effectors is also demonstrated in this study. Overall, this study establishes a strong foundation for further studies elucidating the pathogenic mechanisms employed by *P. capsici* infecting black pepper and for developing effective disease management strategies. Future investigations building upon these findings are essential for advancing our understanding of this pathosystem and for implementing targeted approaches to mitigate black pepper foot rot.

## 1 Introduction

Black pepper (*Piper nigrum* L.), rightly dubbed “The King of Spices” (Hu et al., 2019), owes its title to its widespread use, versatility, and historical importance, all of which underscore its significance and prominence in the world of spices. The dried berries, commonly known as peppercorns, are utilized either whole or in powdered form to enhance food flavor and enrich the taste. This spice crop, belonging to the Piperaceae family, is also considered a medicinal plant with potential nutraceutical and pharmaceutical properties (Takooree et al., 2019). It is a perennial woody climber that thrives in humid tropical and subtropical regions. Considered native to the Western Ghats of South India, it is now cultivated in Vietnam, Indonesia, India, Sri Lanka, China, Cambodia, and Malaysia (Hu et al., 2019). Of all the diseases affecting black pepper, the most destructive is the “quick wilt” or “foot rot” disease caused by the oomycete pathogen *Phytophthora capsici* (Hao et al., 2016; Nguyen et al., 2020).

*P. capsici* has a broad host range that includes several important vegetable crops like tomato, pepper, cucumber, eggplant, and, lima beans (Lamour et al., 2012b) and is ranked the world’s fifth most destructive oomycete (Kamoun et al., 2015). Though it has a fungus-like morphology, *Phytophthora* is classified under the Kingdom Chromista, which also includes diatoms and brown algae (Martens et al., 2008; Beakes et al., 2012; Lamour et al., 2012b). *P. capsici* exhibits a hemibiotrophic lifestyle with an initial asymptomatic biotrophic phase followed by a necrotrophic phase during which it kills the host cells (Fawke et al., 2015). Once the soil is infected, controlling *P*. *capsici* becomes challenging as the pathogen persists in the form of oospores or mycelium in the soil and plant remnants. The oospores show resilience to harsh environments enabling the pathogen to survive many years without a host plant (Wang et al., 2020). Like in other susceptible crops, *P. capsici* can infect every part of black pepper, including the roots, stems, leaves, and fruits, and inflict substantial damage resulting in significant yield losses in black pepper (Hao et al., 2016; Sanogo et al., 2023). Symptoms on leaves appear as progressing black water-soaked lesions with fimbriate margins (Guha Roy, 2015). Since no varieties of black pepper are entirely resistant to *P. capsici*, farmers typically rely on chemical fungicides to control the spread of this disease (Nguyen et al., 2020; Suraby et al., 2020). Besides the toxicity caused by these chemicals on farmers as well as on the ecosystem, this rapidly evolving pathogen is developing resistance to these treatments thereby reducing their effectiveness as fungicides (Parra and Ristaino, 2001).

Plants, although facing constant challenges from various microbial parasites and lacking an adaptive immune system, rely on their innate immune system for defense (Jones and Dangl, 2006; Spoel and Dong, 2012). Invaders, whether they successfully colonize the host or not, are recognized by host cell receptors through Microbe-Associated Molecular Patterns (MAMPs), Damage-Associated Molecular Patterns (DAMPs), and effectors. The successful pathogens or mutualists, have evolved strategies to evade detection, such as sequence diversification, post-translational modification, or loss of ligands, or by directly subverting host immunity through the deployment of biochemically active effectors (Cook et al., 2015). The proteome changes associated with the responses of black pepper against *P. capsici* infection revealed that the innate immunity of black pepper is the key mechanism targeted and manipulated by *P. capsici* (Mahadevan et al., 2016). Oomycete effectors have emerged as valuable tools for investigating plant immune responses and studying host-pathogen evolution (Judelson and Ah-Fong, 2019). They are pathogen molecules that alter the structure and function of host cells, aiding in infection and/or eliciting defense responses. Based on the target site within the host, oomycete effectors are broadly classified into cytoplasmic and apoplastic types (Kamoun, 2006). Functional genomic approaches are essential for identifying pathogen effector genes that trigger plant cellular and molecular responses (Kanneganti et al., 2006). While traditional methods of heterologous gene expression involve stable transgenic plants, which are time-consuming, transient expression systems like agroinfiltration offer a rapid and straightforward alternative. These systems are widely used for studying plant-microbe interactions, allowing high-throughput analysis without the limitations of chromosomal positional effects (Fischer et al., 1999; Kanneganti et al., 2006).

Recently, transcriptome-based RNA-seq studies have been widely used for analyzing key genes and underlying mechanisms involved in host-pathogen interactions (Tu et al., 2023). Despite being an important spice crop, understanding host-pathogen interaction from the pathogen’s perspective is limited in the *P. capsici*-black pepper pathosystem. The present study utilizes an RNA-seq approach to study the changes in gene expression of *P. capsici* during its interaction with black pepper and demonstrates the feasibility of using agroinfiltration-mediated transient expression assays in black pepper to investigate effector roles. These understandings will set the stage for the development of better strategies to control the disease.

## 2 Materials and methods

### 2.1 Plant and pathogen growth conditions, inoculation

Black pepper variety Panniyur-I, confirmed by the herbarium curator and deposited as herbarium specimen with voucher number KUBH 11328 at the Department of Botany, University of Kerala, Thiruvananthapuram, susceptible to *P. capsici* were grown in the greenhouse at Rajiv Gandhi Centre for Biotechnology, Thiruvananthapuram, and used for the current study. The *P. capsici* isolate RGCB0451 was continuously maintained by routine sub-culturing on Potato Dextrose Agar (PDA) media (Himedia) at a constant temperature of 28°C in the absence of light. The second and third true leaves of black pepper were inoculated with 4 days old *P. capsici* mycelia following the sandwiched inoculation method outlined by Chen et al. (2007) with minor modifications. In brief, *P. capsici* was grown on PDA medium covered with a sterile cellophane membrane, purchased from a local store, that enables nutrient access and facilitates easy separation of mycelia (Schumann et al., 2013). The four-day-old mycelium was scraped off with a sterile scalpel and carefully sandwiched between the adaxial surfaces of two detached black pepper leaves after pricking the leaves with a sterile needle. The inoculated leaves were placed on a moistened Whatman filter paper inside a square petri plate and sealed with parafilm to preserve humidity. Sampling of mycelia was conducted at 1.5, 3, 6, 12, 24, and 48 h post inoculation (hpi) for RNA isolation. The early time points, from 1.5 hpi to 12 hpi, were chosen to capture gene expression and infection dynamics during the initial stages of infection. Only mycelia were harvested at these early time points, with approximately 200 mg collected from each time point. Mycelia harvested at 24 and 48 hpi were collected along with the infected leaf material, with equal portions of mycelia and leaf tissue (about 100 mg each), making a total of 200 mg. For each time point, three biological replicates were collected. Samples were immediately frozen in liquid nitrogen and used for RNA isolation.

### 2.2 Lesion scoring and microscopic evaluation

For pathogen lesion area scoring and trypan blue staining, the mycelial agar disk inoculation method was performed as described in Krishnan et al. (2015). Trypan blue staining was used for microscopic visualization of the progression of *P. capsici* growth in black pepper leaves according to the protocol described in Chung et al. (2010). Microscopic images were captured in Nikon Eclipse-Ni microscope under 10x magnification. The diameter of lesions was measured on 16 leaves inoculated with *P. capsici*, and the area was calculated using the formula $A=\pi r^{2}$ , where r represents the radius of the lesion.

### 2.3 RNA extraction, library preparation, and sequencing

RNA extraction was carried out using the RNeasy Plant Mini Kit (Qiagen) according to the manufacturer’s instructions. The frozen samples were ground to a fine powder in liquid nitrogen using a mortar and pestle. The powdered tissue was then processed according to the kit’s protocol to extract total RNA. The RNA from 1.5, 3, and 6 hpi mycelia were pooled as early infection time point (PcPn_E), and 12, 24, and 48 hpi as late infection time point (PcPn_L). RNA from four-day-old mycelia directly from the PDA medium was used as the control group (Pc). The quantification of RNA was performed using NanoVue, and the quality of RNA was assessed through RNA ScreenTape (Agilent) as well as 1.5% Agarose-Formaldehyde gel electrophoresis. For mRNA enrichment, 250 ng of total RNA underwent purification using the NEBNext Poly(A) mRNA Magnetic Isolation Module (New England Biolabs), following the manufacturer’s protocol. The enriched mRNAs were then utilized for library preparation employing the NEBNext^®^ Ultra^™^ II RNA Library Prep Kit for Illumina (New England Biolabs). In brief, the enriched mRNAs were primed with NEBNext Random Primers and chemically fragmented in a magnesium-based buffer at 94°C for 7 min, resulting in inserts of approximately 200 nucleotides. Subsequently, the fragmented mRNAs were reverse-transcribed to form cDNA, and the first-strand cDNA reactions were converted to double-stranded DNA (dS DNA) using NEBNext second strand synthesis reaction buffer and enzyme mix. After adapter ligation, the cDNA was subjected to 7 cycles of PCR amplification using NEBNext Ultra II Q5 master mix, along with NEBNext^®^ Multiplex Oligos for Illumina. The final cDNA library was then eluted and quantified in a Qubit 3 Fluorometer (Life Technologies). The quality assessment of the library was conducted using the Agilent D5000 ScreenTape System (Agilent) and Agilent D1000 ScreenTape System (Agilent) on a 4150 TapeStation System (Agilent). The sequencing of the prepared libraries was performed on the Illumina NovaSeq 6000 platform using paired-end 150 bp reads. Sequence reads underwent preprocessing to eliminate adapter sequences and low-quality bases using fastp v0.20 (Chen S. et al., 2018). The quality-controlled reads were aligned to the indexed *P. capsici* genome^1^ (Lamour et al., 2012a) using the STAR v2 aligner (Dobin et al., 2013). Gene-level expression values were obtained as read counts using the feature-counts software (Liao et al., 2014). Functional information was obtained through gene annotation performed using the NCBI non-redundant database, UniProt database, and Gene Ontology. A clustering analysis was conducted using the Clust (Abu-Jamous and Kelly, 2018) tool to identify infection-responsive genes that showed coordinated expression and cluster transcripts with similar accumulation profiles. The analysis utilized the k-means clustering method, with parameters set to a tightness level of 10 and a Q3s outliers threshold of 2.0. The RNA-seq data were normalized using the relative log expression (RLE) function in the DESeq2 package. Differential expression analysis was conducted with DESeq2 (Love et al., 2014) to identify differentially expressed genes (DEGs). Benjamini and Hochberg’s method of false discovery rate (FDR) correction was used to adjust the *p-*values. Genes with a log_2_ fold change ≥1 or ≤−1 and an FDR less than 0.05 were considered significant. Three pairwise comparisons were performed to identify DEGs: between the control group (Pc) and the early infection time points (PcPn_E), between the control group (Pc) and the late infection time points (PcPn_L), and between the early (PcPn_E) and late infection time points (PcPn_L). Overrepresentation analysis for Biological Process, Molecular Function, and Cellular Component was carried out using the ClusterProfiler R Bioconductor package (Yu et al., 2012). Gene Ontology (GO) terms with multiple-test-adjusted *p*-values ≤ 0.05 were deemed significant.

### 2.4 RT-qPCR for validation of RNA-seq data

Six *P. capsici* effector genes were selected due to their significant differential expression during the infection process to validate the expression changes observed in RNA-seq analysis using RT-qPCR. The sequences of these effectors were retrieved from the JGI genome portal.^2^ The primers were designed using the Primer 3 Plus bioinformatics tool.^3^ Melt curve analysis was conducted after the PCR phase to confirm the primer specificity and absence of primer dimers. Further details regarding the primers, including their sequences and amplicon size are provided in Supplementary Table S1. Three *P. capsici* reference genes *ubc*, *ws21*, and *ef1α* were used for the normalization of gene expression in RT-qPCR (Vijayakumar and Sakuntala, 2024). The cDNA synthesis was carried out in a 20 μL reaction volume containing 1 μg RNA using the PrimeScript™ RT reagent kit (TaKaRa, Japan). The reaction mix was incubated at 37°C for 15 min, followed by a heat treatment at 85°C for 5 s. Diluted aliquots of cDNA were used as templates for RT-qPCR assay. RT-qPCR was performed on QuantStudio^™^ 5 Real-Time PCR system (Applied Biosystems, United States) employing the TB Green^®^ Premix Ex Taq^™^ II (Tli RNaseH Plus) kit (TaKaRa, Japan). The reaction mixture, with a total volume of 20 μL, comprised of 10 μL TB Green Premix Ex Taq^™^ II (2X), 0.4 μL ROX Reference Dye II, 2 μL cDNA template, 0.4 μM each of forward and reverse primers and 6 μL nuclease-free water. The cycling conditions for RT-qPCR involved an initial denaturation step at 95°C for 10 min, followed by 40 cycles of denaturation at 95°C for 15 s and annealing at 60°C for 30 s. The assay was performed for three biological replicates with technical triplicates and no template controls. Relative fold change was analyzed using the Pfaffl method (Pfaffl, 2001).

### 2.5 Functional analysis of *PcNLP6* through agroinfiltration in black pepper

*Nicotiana benthamiana* plants were grown under a 24°C temperature regime with a 16-h light and 8-h dark cycle until they reached the eight-leaf stage. The binary vector pCAMBIA1305.2, purchased from Abcam was used for the study. The *β*-glucouronidase (GUS) gene in the pCAMBIA1305.2 was replaced by the *P. capsici* apoplastic effector *PcNLP6* or its site-directed mutants. Three amino acids -K214, H223, and, E228 -from the conserved domain of *PcNLP6* were replaced by alanine through site-directed mutagenesis. The genes were synthesized and delivered in pCAMBIA 1305.2 by GeneArt Custom Gene Synthesis, ThermoFisher Scientific to generate pCAMBIA 1305.2:: NLP6, pCAMBIA 1305.2:: NLP6 ^K241A^, pCAMBIA 1305.2:: NLP6 ^H223A^, and, pCAMBIA 1305.2: NLP6 ^E228A^. The constructs were introduced into *A. tumefaciens* strain GV3103 by the freeze–thaw method (Hofgen and Willmitzer, 1988). The transient expression assay followed the process outlined by Kanneganti et al. (2006). The same method was employed for the transient expression study of *PcNLP6* in black pepper on the second and third mature detached leaves. Since transient expression in black pepper has not been previously reported, initially a GUS histochemical assay (Jefferson et al., 1987) was performed following the agroinfiltration of the empty vector pCAMBIA1305.2-GUS before infiltrating them with wild-type *PcNLP6* and its mutants. Cell death was detected using trypan blue staining (Ma et al., 2012).

### 2.6 Statistical analysis and visual representation

Statistical analysis and violin plot for depicting lesion area were performed using GraphPad Prism software (v 9.5.1). Heatmaps were generated using TBtools-II v2.083 (Chen et al., 2023). Venn diagrams were constructed using Venny 2.1^4^ (Oliveros, 2007). GO enrichment analysis bubble plots were generated using SRplots (Tang et al., 2023).

## 3 Results

### 3.1 Disease development

**Figure 1:**
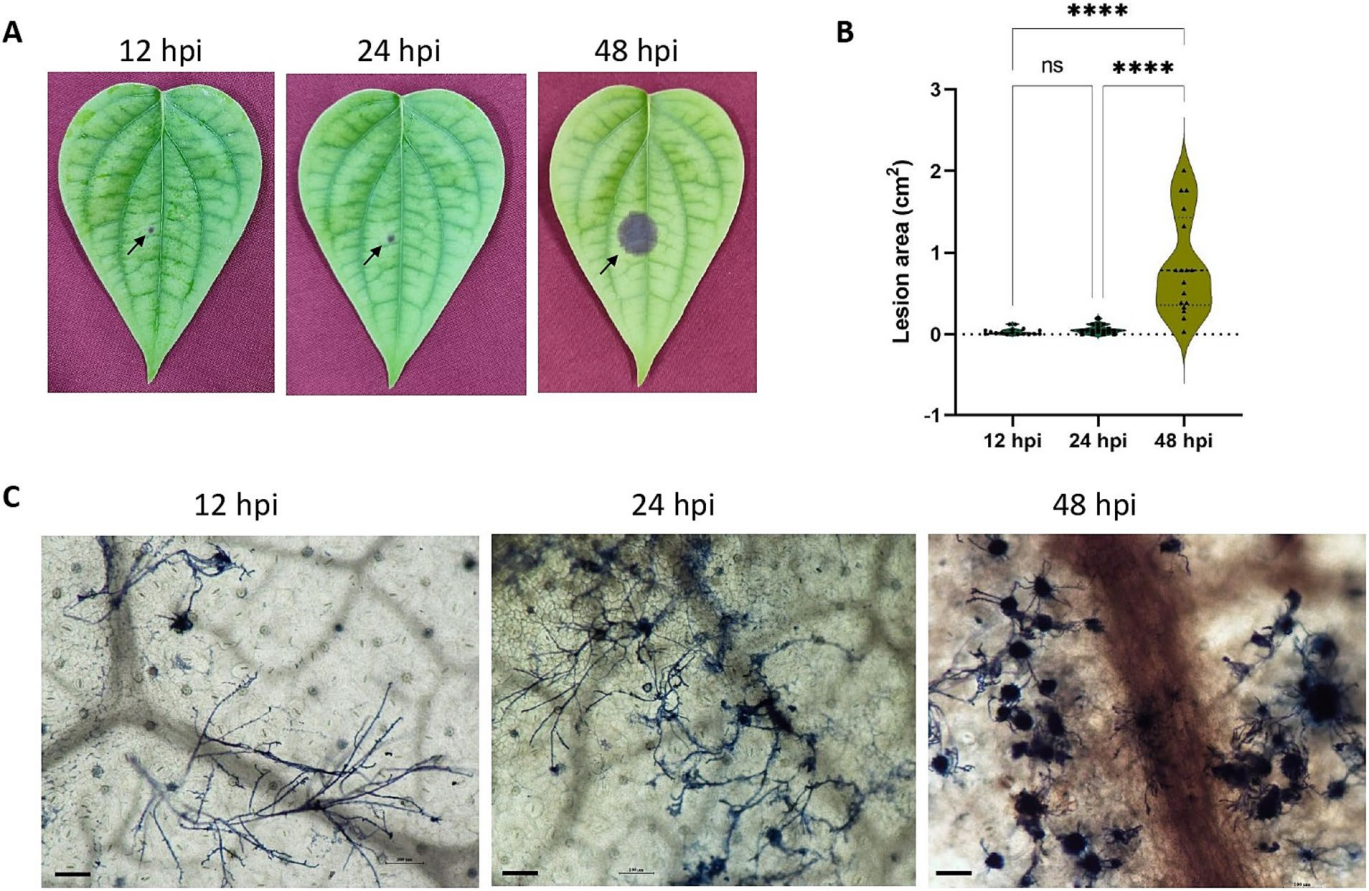
**(A)** *P. capsici* infection progression in black pepper leaves at 12 hpi, 24 hpi, and 48 hpi. **(B)** Violin plot depicting the lesion area (cm^2^) in 12 hpi, 24 hpi and 48 hpi. Statistical analysis was performed using Kruskal-Wallis followed by Dunn’s multiple comparisons test, **** represents *p* value < 0.0001. **(C)** Trypan blue staining image depicting the microscopic growth of *P. capsici* in black pepper leaves at 12 hpi, 24 hpi, and 48 hpi under 10× magnification. Scale bar: 100 μm.

Lesions were observable within 12 hpi onwards (Figure 1A) and showed significant expansion in area by 48 hpi (Figure 1B). Microscopic observations showed the spread of filamentous hyphae from 12 hpi to massive hyphal growth by 48hpi (Figure 1C).

### 3.2 RNA sequencing

**Figure 2:**
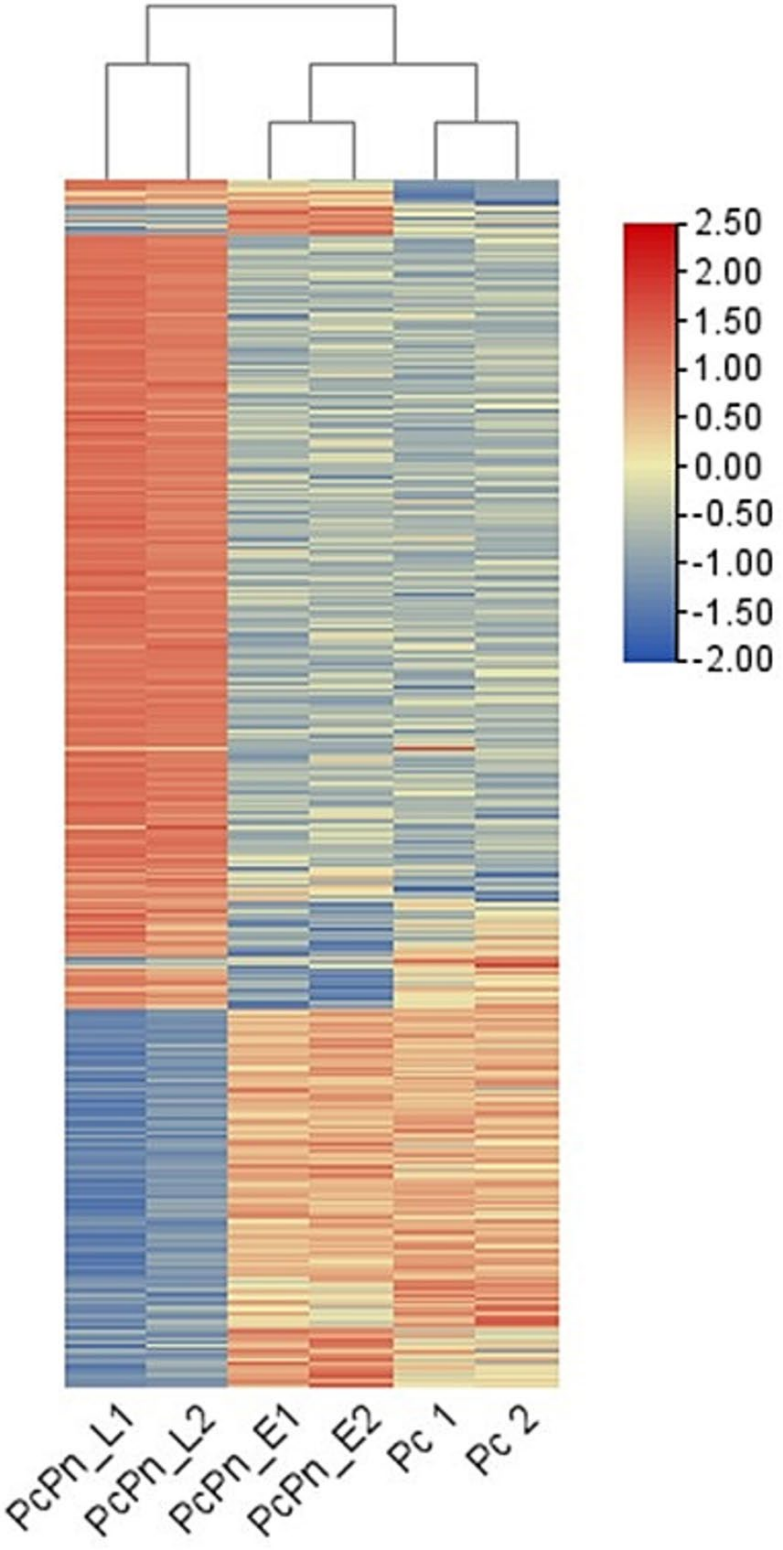
Heatmap of differentially expressed genes. Each row shows the normalized expression count on log_2_ scale. The scale bar shows the expression level with blue and red colors indicating low and high expression. The heatmap was generated using TBtools-II v2.083.

RNA-seq yielded 243 million reads in total, with Q 30 percentage exceeding 94.7%. Following quality control, 88.3% of the reads, totaling 214 million, were retained. The Pc, PcPn_E, and PcPn_L libraries contained an average of 38,555,015, 35,585,660, and 33,167,604 cleaned reads, respectively (Table 1). Among these, 20,840,310 to 41,997,292 reads were successfully mapped to the reference genome of *P. capsici*, leading to the detection of 14,298 putative genes after normalization of the expression counts (Supplementary Table S2). All the raw reads were uploaded to NCBI under the BioProject number PRJNA932159. The holistic expression of all genes was visualized using a heatmap wherein three clusters were obtained with the replicates of each group coming under the same cluster (Figure 2).

**Table 1:** Summary of RNA sequencing results obtained from *P. capsici* infecting black pepper.

Sample	Total reads	Mapped reads	Mapped reads %	Unmapped reads	Unmapped reads %	Uniquely mapped	Uniquely mapped %
Pc 1	35,010,678	34,930,520	99.77	80,158	0.23	33,175,616	94.76
Pc 2	42,099,352	41,997,292	99.76	102,060	0.24	39,911,002	94.8
PcPn_E 1	33,602,602	33,510,328	99.73	92,274	0.27	31,678,208	94.27
PcPn_E 2	37,568,718	37,461,656	99.72	107,062	0.28	35,264,012	93.87
PcPn_L 1	26,479,914	20,840,310	78.7	5,639,604	21.3	15,819,210	59.74
PcPn_L 2	39,855,294	32,963,478	82.71	6,891,816	17.29	26,560,484	66.64

### 3.3 Co-expression clustering analysis

**Figure 3:**
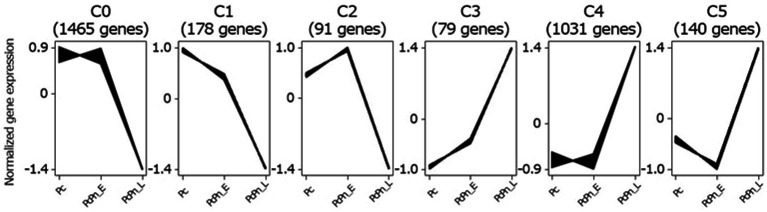
Clustering of co-expressed infection-responsive genes in *P. capsici* based on their normalized expression values using the Clust algorithm. The numbers in parentheses represents the number of genes in each cluster.

To identify transcripts with similar accumulation patterns during the infection process, we performed a clustering analysis on all infection-responsive transcripts using the Clust tool (Abu-Jamous and Kelly, 2018) and identified six distinct clusters (Figure 3). The largest cluster, C0, comprising 1,465 genes, exhibited a decrease in gene expression between PcPn_E and PcPn_L. The second-largest cluster, C4, containing 1,031 genes, displayed an increase in gene expression between PcPn_E and PcPn_L. Cluster C1 showed continuous repression from Pc to PcPn_L, while Cluster C2 demonstrated an initial induction followed by repression. In the cluster C3, genes showed an initial induction that persisted into PcPn_L, and cluster C5 displayed repression at PcPn_E, followed by induction in PcPn_L.

### 3.4 Differentially expressed gene analysis in *Phytophthora capsici*

**Figure 4:**
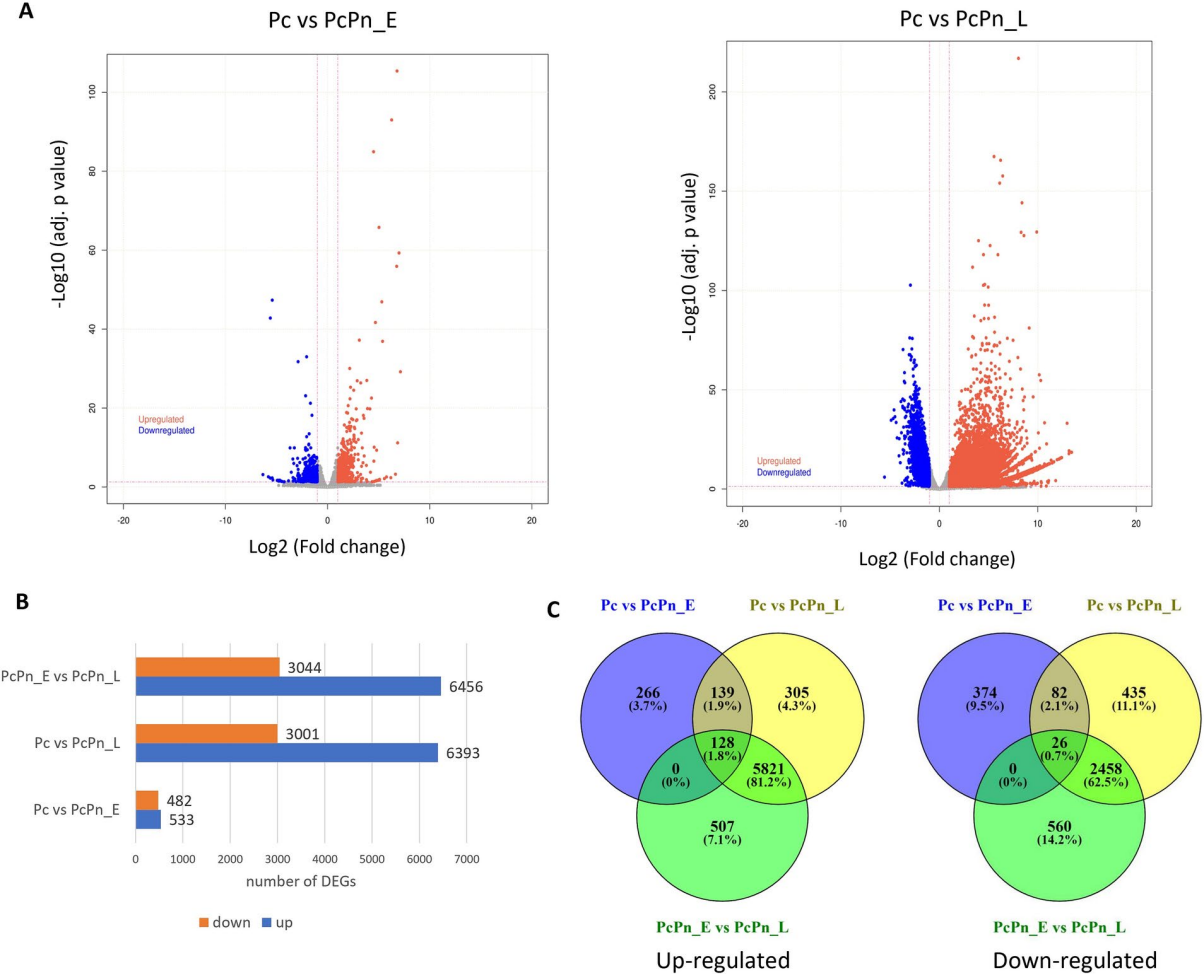
**(A)** Volcano plots depicting the proportion of DEGs in the pairwise comparisons Pc vs. PcPn_E and Pc vs. PcPn_L. Significantly upregulated and downregulated genes are represented by red and blue dots, respectively, and gray dots represent genes without a significant differential expression. **(B)** Bar plot depicting the number of DEGs in each pairwise comparison. **(C)** Venn diagram represents the summary of the overlap between DEGs.

To understand the difference in expression patterns of genes during the infection hours with the control group, a differential expression analysis was conducted among the libraries using DESeq2 and volcano plots were generated to visualize the distribution of genes that showed significant differential expression (Figure 4A). The lists of all differentially expressed genes are provided in Supplementary Tables S3–S5. In the Pc vs. PcPn_E, Pc vs. PcPn_L, and, PcPn_E vs. PcPn_L comparisons, 533, 6,393, and, 6,456 genes were upregulated, while 482, 3,001, and, 3,044 genes were downregulated, respectively (Figure 4B). To elucidate uniquely and shared expressed genes among the three groups, a Venn diagram was generated (Figure 4C). In comparison to the control group (Pc), 266 and 305 genes were uniquely upregulated, and, 374 and 435 genes were uniquely downregulated in the PcPn_E and PcPn_L samples, respectively. Moreover, 139 upregulated and 82 downregulated genes were shared between the PcPn_E and PcPn_L samples when compared to the control group. Furthermore, 507 upregulated and 560 downregulated genes were uniquely expressed in PcPn_L compared to PcPn_E. Lastly, 5,821 upregulated and 2,458 downregulated genes were specific to PcPn_L when compared to both Pc and PcPn_E. The greatest number of DEGs were between PcPn_E and PcPn_L. The upregulated and downregulated genes in the PcPn_E and PcPn_L samples compared to the control (Pc) provide insight into the specific strategies utilized by *P. capsici* to adapt and thrive in the host environment, which could be further explored for their roles in the infection process.

### 3.5 Validation of RNA-seq data

**Figure 5:**
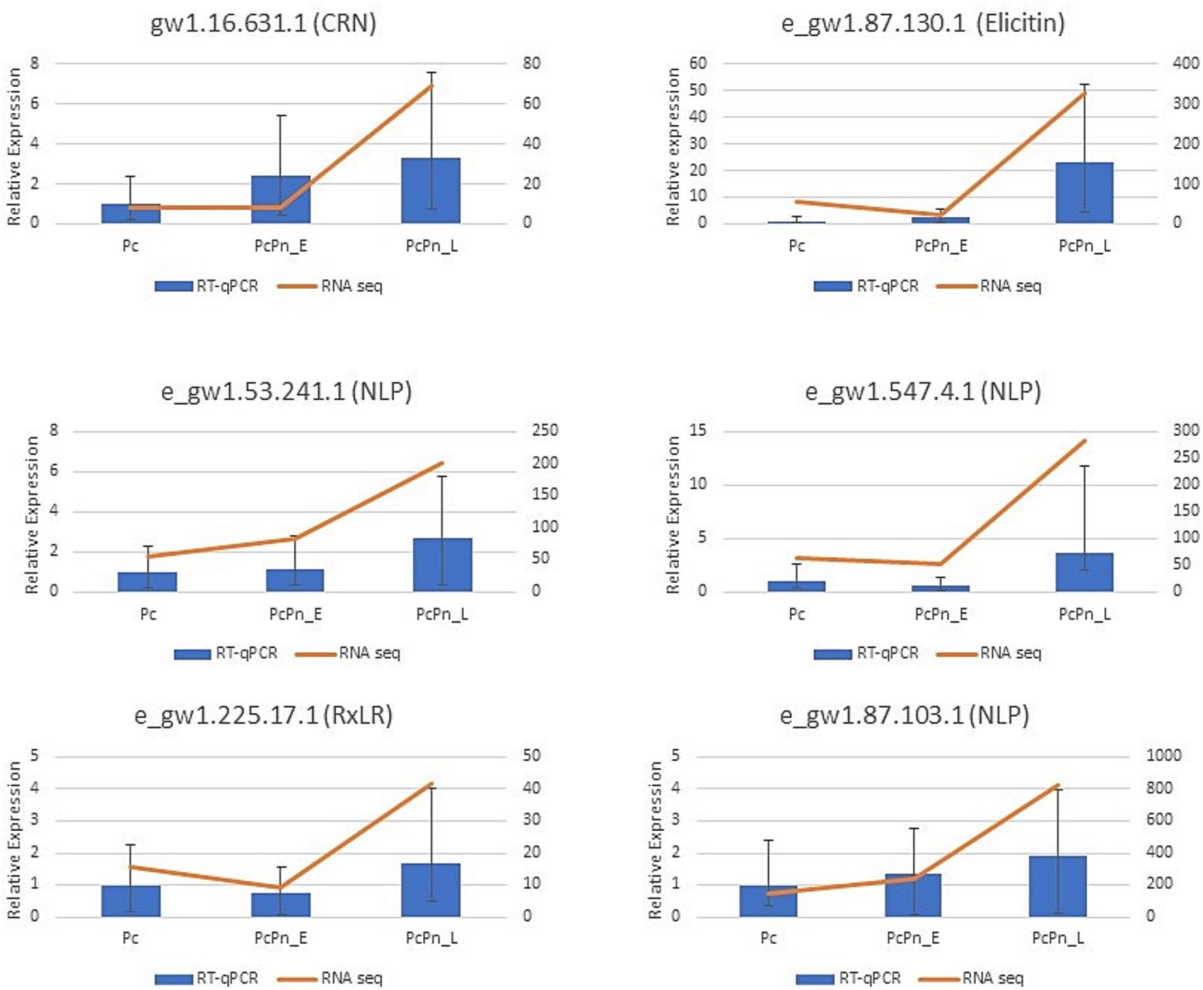
RT-qPCR results indicate the relative expression of six effector genes in Pc, PcPn_E, and PcPn_L to validate the RNA-seq analysis results. Error bars represent the standard error.

RT-qPCR analysis was conducted to validate the expression levels of genes obtained in RNA sequencing. The expressions of six effector genes from the DEG list in this study were analyzed, alongside three internal control genes -*ubc, ws21, and ef1α* (Vijayakumar and Sakuntala, 2024). The obtained results were consistent with the transcriptome data (Figure 5). The expression levels of transcripts corresponding to NLPs (e_gw1.53.241.1 and e_gw1.87.103.1), CRN (gw1.16.631.1), and elicitin (e_gw1.87.130.1) exhibited a minor increase in PcPn_E, followed by a substantial induction in PcPn_L. Conversely, the transcripts of RxLR (e_gw1.225.17.1) and NLP (e_gw1.547.4.1) showed a slight decrease in expression in PcPn_E but were up-regulated in PcPn_L.

### 3.6 Gene ontology enrichment analysis

**Figure 6:**
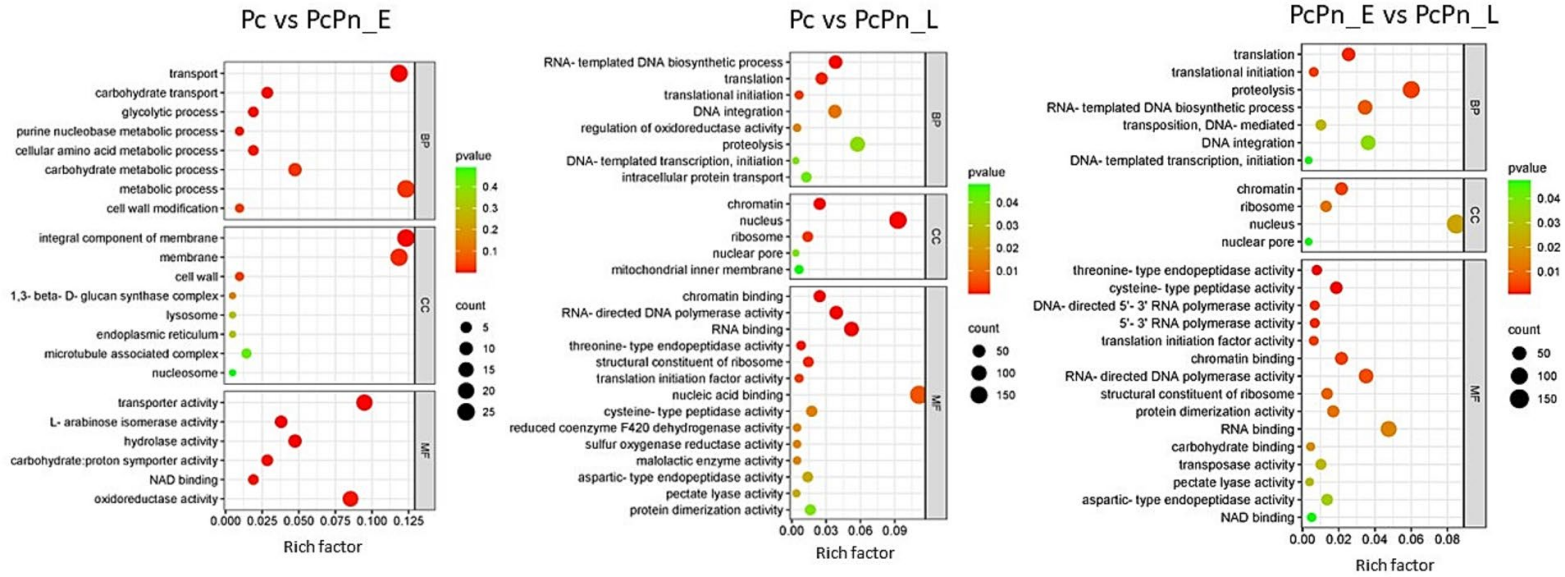
Bubble plots depicting the enriched GO terms in Pc vs. PcPn_E, Pc vs. PcPn_L, and PcPn_E vs. PcPn_L.

To categorize the DEGs based on their associated Biological Processes (BP), Molecular Functions (MF), and, Cellular Components (CC), a gene ontology analysis was carried out. Among the differentially expressed genes, the top GO entries with the most significant enrichment were selected in three categories to represent in the bubble plots (Figure 6). In Pc vs. PcPn_E, the differentially expressed genes showed enrichment in the BP terms transport (GO:0006810), carbohydrate transport (GO:0008643), glycolytic process (GO:0006096), purine nucleobase metabolic process (GO:0006144), cellular amino acid metabolic process (GO:0006520), and, carbohydrate metabolic process (GO:0005975). In CC they were enriched in integral component of membrane (GO:0016021) and membrane (GO:0016020), and in MF transporter activity (GO:0005215), L-arabinose isomerase activity (GO:0008733), hydrolase activity (GO:0004553), carbohydrate: proton symporter activity (GO:0005351), NAD binding (GO:0051287), and, oxidoreductase activity (GO:0016491) terms were enriched. Translation (GO:0006412), translation initiation (GO:0006413), RNA-templated DNA biosynthetic processes (GO:0006278), proteolysis (GO:0006508), and, DNA integration (GO:0015074) in BP, chromatin (GO:0000785), nucleus (GO:0005634), nuclear pore (GO:0005643), and, ribosome (GO:0005840) in CC and, chromatin binding (GO:0003682), RNA binding (GO:0003723), RNA-directed DNA polymerase activity (GO:0003964), threonine type endopeptidase activity (GO:0004298), and, pectate lyase activity (GO:0030570) in MF were some of the significantly enriched terms common to Pc vs. PcPn_L and PcPn_E vs. PcPn_L.

### 3.7 Transient expression of *PcNLP6* induces cell death in black pepper

**Figure 7:**
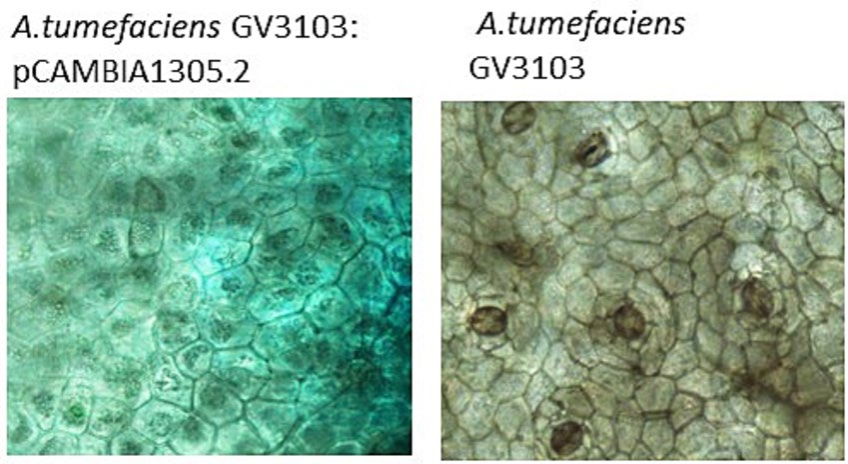
Histochemical GUS staining in black pepper leaves at 2 days post agroinfiltration.

**Figure 8:**
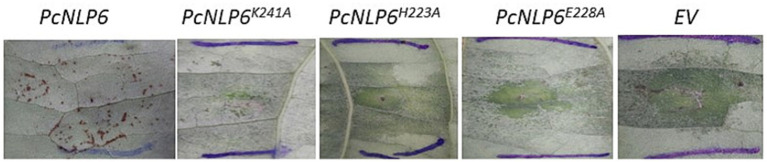
Agroinfiltrated black pepper leaves at 4 days post infiltration.

As reported in Feng et al. (2014), the wildtype *PcNLP6* induced cell death in *N. benthamiana* whereas none of the mutants exhibited this effect (Supplementary Figure S1). In black pepper leaves infiltrated with *A. tumefaciens* GV3103 harboring pCAMBIA1305.2, GUS staining assay showed blue colouration as early as 2 days post infiltration whereas no color was observed in black pepper leaves infiltrated with *A. tumefaciens* GV3103 alone (Figure 7). The presence of GUS gene transcripts was also confirmed by RT-PCR (Supplementary Figure S2). When infiltrated with wildtype *PcNLP6*, the necrosis pattern appeared as small brown spots in the infiltrated region, which differed from the pattern observed in *N. benthamiana* (Figure 8). Trypan blue staining of these regions in black pepper confirmed the cell death activity (Supplementary Figure S3). These brown spots were absent in the mutants as well as in the empty vector control (Figure 8).

## 4 Discussion

Black pepper holds economic significance as a spice crop but encounters cultivation and production challenges stemming from quick wilt disease caused by *P. capsici*. However, the molecular mechanisms underlying *P. capsici* infection in black pepper remain elusive. RNA-seq is a promising approach in scenarios like this, to set the platform for obtaining detailed insights on the molecular intricacies of the pathogen infection strategy. Our study aims to elucidate this mechanism by identifying the transcriptional alterations occurring in *P. capsici* during its invasion of black pepper leaves by comparing the axenic culture mycelium to mycelium at various time points of infecting the host. The inoculation method employed in this study, derived from Chen et al. (2007), is extensively utilized in *Phytophthora* research (Yang et al., 2013; Yin et al., 2013; Li et al., 2014; Ma et al., 2018). It involved a sandwiched mycelial approach for artificially inoculating *P. capsici* on black pepper leaves. This technique facilitates the exploration of transcriptional alterations during the early stages of infection, addressing the challenge of obtaining adequate pathogenic RNA from leaf samples in the initial hours, as encountered with zoospore inoculation (Ma et al., 2018). In the pathogen inoculation assay, symptoms were observable to the naked eye within 12 h post-inoculation (Figure 1), similar to the findings of Mahadevan et al. (2016).

**Figure 9:**
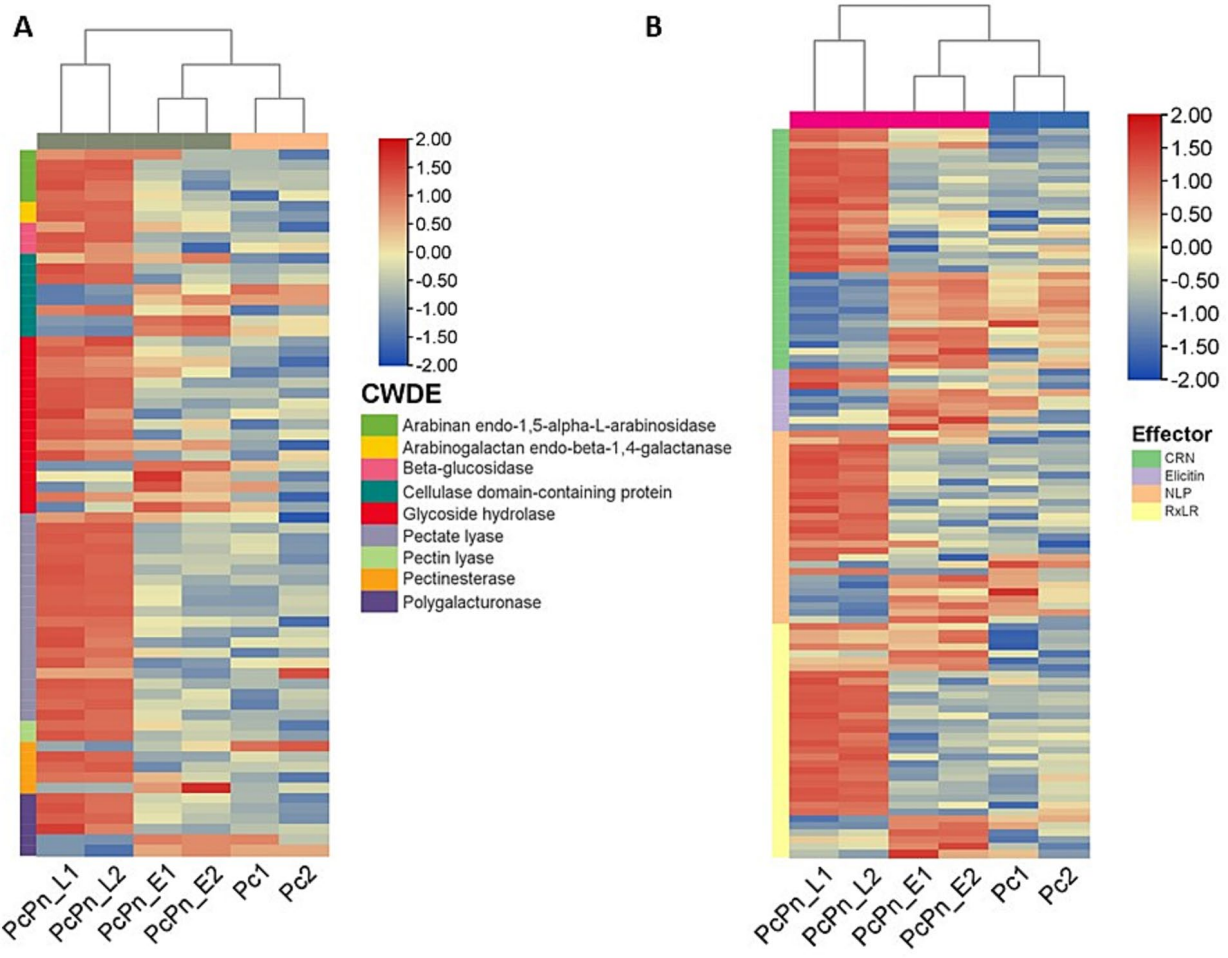
Heatmaps of differentially expressed genes **(A)** cell wall degrading enzymes **(B)** effectors. Each row shows the normalized expression count on log_2_ scale. The scale bar shows the expression level with blue and red colors indicating low and high expression. The heatmap was generated using TBtools-II v2.083.

Cell wall degrading enzymes (CWDEs) potentially serve to dismantle plant cell walls at penetration sites and may also alter the structure of *Phytophthora* cell walls at these intimate contact points, facilitating more efficient exchange with host cells (Boevink et al., 2020). During infection, a wide range of CWDE family members are expressed, although specific members may be expressed in different lifecycle stages, hosts, or tissue types (Attard et al., 2014; Blackman et al., 2015). The cellulase class of genes showed marginal upregulation, in PcPn_E compared to Pc, whereas most of them exhibited downregulation in PcPn_L. Conversely, in *P. parasitica* pectinases are predominantly expressed in the early hours, and cellulases and hemicellulases dominate later on (Blackman et al., 2015). Understanding the expression pattern of CWDEs aids in elucidating the role of these enzymes in pathogen virulence. In our study, about 20 pectate lyase (PL) genes and 12 glycoside hydrolase (GH) genes were upregulated in PcPn_L (Figure 9A). Transcript level expression of a PL gene (estExt2_Genewise1.C_PHYCAscaffold_850068) was increased to >300 folds in the PcPn_L library. CWDEs like PLs and GHs are known to be associated with pathogen virulence, inducing cell death and triggering defense responses in plants, specifically, cell death-inducing proteins (CDIPs) have been identified in CWDE families like carbohydrate esterase, glycoside hydrolase, and polysaccharide lyase (Li et al., 2020). Pectate lyases, such as PcPL1, PcPL15, PcPL16, and PcPL20 in *P. capsici* (Fu et al., 2015), and VdPEL1 in *V. dahlia* (Yang et al., 2018), play a critical role in pectin degradation and have been shown to induce cell death in plants. During infection of pepper, PcPL1, PcPL15, PcPL16, and PcPL20 were highly expressed and induced severe cell death in pepper leaves (Fu et al., 2015). GHs, on the other hand, hydrolyze glycosidic bonds and have been associated with cell death induction and defense responses in host and nonhost plants. Approximately 18 GH domain-containing CDIPs have been identified, some of which possess hydrolase activity. Interestingly, while hydrolase activity of certain GH family CDIPs like BcPG2 was required for cell death induction, the enzymatic activity of most GH family CDIPs was not essential for their cell death-inducing effects (Li et al., 2020). Therefore, the significant upregulation of PLs and GHs in PcPn_L may suggest an association with the switch to a necrotrophic lifestyle of *P. capsici* in black pepper infection, avoiding plant cell death during early infection hours, which is consistent with the hemibiotrophic nature of this pathogen. A similar observation was also reported in the study on transcript profiling of *P. capsici* during its interaction with *A. thaliana* (Ma et al., 2018). Additionally, certain polygalacturonases, arabinan endo-1,5-alpha-L-arabinosidase, and arabinogalactan endo-beta-1,4-galactanase were observed to be upregulated in this library. In a previous study exploring the effects of tagatose on *P. cinnamomi*, researchers observed a similar upregulation of arabinan-degrading enzymes that release pentose sugars from polysaccharide side chains and suggested that the organism may metabolize alternative sugars and enhance pentose metabolism to counteract any metabolic impacts of tagatose (Chahed et al., 2021).

*P. capsici* enhances its virulence through the secretion of apoplastic and cytoplasmic effectors. While infecting black pepper, all major classes of effectors—including RxLR, CRNs, NLPs, and elicitins—exhibited significant changes in expression levels (Figure 9B; Table 2). The alterations in the transcriptome were validated by conducting RT-qPCR on a subset of randomly selected effectors from these classes, including RxLRs, NLPs, CRNs, and elicitins.

**Table 2:** Differentially expressed effector genes in *P. capsici* during infection.

Effectors	Pc vs. PcPn_E		Pc vs. PcPn_L		PcPn_E vs. PcPn_L		Total number
	Up	Down	Up	Down	Up	Down	
CRN	5	1	16	8	17	13	35
Elicitin	1	–	3	3	2	6	9
NLP	3	2	17	7	17	6	28
RxLR	11	1	25	2	20	5	34

Necrosis and ethylene-inducing peptide 1 (Nep 1)-like proteins (NLPs) constitute an important apoplastic effector family (Kamoun, 2006) produced by bacteria, fungi, and oomycete, with a notable expansion in oomycetes. They have been identified to induce necrosis or stimulate defense responses in dicots or act as noncytotoxic proteins with unknown functions (Seidl and Van Den Ackerveken, 2019). Pirc et al. (2022) recently elucidated the mode of membrane disruption of a cytolytic NLP from *Pythium aphanidermatum*, involving its initial association with membrane lipid receptors, GIPCs, followed by the formation of transient pores in the plant plasma membrane thereby allowing the passage of small molecules. A study by Chen X. R. et al. (2018) revealed 65 potential NLP genes in the *P. capsici* genome. In the present study, a total of 28 NLPs were significantly upregulated in *P. capsici* while infecting black pepper. NLP7 (e_gw1.14.843.1) showed five-fold upregulation in the PcPn_E. NPP11 (e_gw1.477.2.1) was upregulated by more than 2000 folds and NLP6 (e_gw1.39.419.1) by more than 100 folds in the PcPn_L sample. NLP6 (e_gw1.39.419.1) that showed more than 100-fold upregulation in PcPn_L, has been reported to show strong upregulation in *C. annuum* infection, and also contributes to necrotic lesion development in *C. annuum* and *N. benthamiana* leaves (Feng et al., 2014). A total of 9 elicitins, which are another important apoplastic effector group showed differential expression in which 1 was upregulated in PcPn_E, 6 downregulated, and 2 upregulated in PcPn_L. Several small cysteine-rich (SCR) proteins containing the PcF domain (Pfam PF09461) have been identified across various oomycete species. A recent investigation by Zhou et al. (2023) revealed that a PcF/SCR effector within *P. capsici*, SCR82, serves dual roles as both a plant defense elicitor and a virulence factor. Using a combination of transient expression and stable gene silencing methods, Chen et al. (2016) illustrated the significance of the *P. cactorum* PcF/SCR protein SCR96 in plant pathogenicity and oxidative stress tolerance, highlighting its role in inducing programmed cell death (PCD) in plants and enhancing tolerance to oxidative stress. In our study, a PcF gene in the Pfam domain PF09461 exhibited a 140-fold upregulation in PcPn_E and a 55-fold upregulation in PcPn_L.

RxLRs are cytoplasmic effectors that share a common structural feature of an alpha-helical fold formed by tryptophan and tyrosine motifs, and a conserved N terminal Arg-x-Leu-Arg motif for translocation into the host cell (Birch et al., 2006; Boutemy et al., 2011). They are known to suppress plant immunity (Fan et al., 2018) as well as affect protein degradation and stability (Bos et al., 2010), autophagy (Dagdas et al., 2016), kinase and phosphatase signaling (King et al., 2014; Boevink et al., 2016), and brassinosteroid hormone signaling (Saunders et al., 2012). Recently, a study on *P. capsici* RxLR effector CRISIS2 reported that its transcripts were dramatically induced at 6hpi, correlating its role in regulating cell death by suppressing the activity of plasma membrane H+ -ATPase in *N. benthamiana* (Seo et al., 2023). In PcPn_E the highest number of effector representation was from the RXLR family. Eleven RXLRs were upregulated within 6hpi, 20 RXLRs upregulated by 48 hpi, and overall, 34 RXLRs were differentially expressed during the infection hours (Figure 9B).

CRNs (Crinkling and necrosis inducing) represent another prominent cytoplasmic effector group characterized by an LFLAK motif at their N-terminus (Schornack et al., 2010). In the context of the *P. capsici-*black pepper interaction examined in this study, a total of 35 CRNs exhibited significant changes in expression levels over the course of infection. Specifically, at 6 h post-infection, only five CRNs showed upregulation, while compared to PcPn_E, 17 were upregulated and 13 were downregulated in PcPn_L. This stands in contrast to a previous study focusing on pre-infection stages of *P. capsici*, which reported differential expression of only 13 CRNs (Chen et al., 2013), underscoring the significance of CRNs during infection.

GO terms related to transport, carbohydrate transport, and the glycolytic process were enriched in PcPn_E. All genes related to glycolysis within the GO term “glycolytic process” showed upregulation in PcPn_E (Figure 6), indicating the utilization of glucose for ATP production. Genes associated with carbohydrate transport and carbohydrate metabolic processes were also upregulated. Whereas, the genes that encoded major glycolytic enzymes among the DEGs were significantly downregulated in the PcPn_L. This could be due to the utilization of host derived compounds as metabolic substrates as mentioned in Judelson et al. (2008). Phosphoglycerate dehydrogenase, identified as a glycolytic enzyme with an atypical mitochondrial location in *Phytophthora* (Abrahamian et al., 2017) was also present within the DEGs. The glycolytic pathway in *Phytophthora* differs significantly from that of other organisms due to the compartmentalization of several glycolytic enzymes into mitochondria and possible neofunctionalization of enzymes. The capability of these organisms to thrive on diverse plant tissues, characterized by varying nutrient concentrations and compositions, as well as on artificial media, suggests considerable metabolic adaptability. The mechanisms behind this flexibility have started to be revealed to some extent through the advancement of -omics initiatives (Kuhn et al., 2022). In PcPn_E, the GO-enriched terms within the Molecular Function category included “transporter activity” and “hydrolase activity.” These terms mainly comprised genes belonging to the Major Facilitator Superfamily and cellulase, respectively, all of which showed upregulation.

Being involved in detoxification processes and essential for nutrient acquisition, organ growth, and development, the ATP-binding cassette (ABC) transporters may protect *P. capsici* from natural toxic compounds produced by susceptible hosts. The gene family of ABC transporters has significantly expanded in the most aggressive isolates of *P. capsici*, suggesting that they also play a role as virulence-associated effectors during *P. capsici* evolution, akin to RxLR and CRN effectors (Lee et al., 2021). Approximately 32 ABC transporters exhibited upregulation in PcPn_L compared to Pc, with the majority showing upregulation by more than five-fold. This suggests that during the interaction between *P. capsici* and black pepper, ABC transporters are significantly induced between 12 to 48 h post-infection, rather than within the initial 6 h post-infection. Transcriptional activation of genes encoding ABC transporters could potentially be associated with both detoxification processes and the secretion of virulence factors (Abou Ammar et al., 2013). Plants release toxic compounds like phytoanticipins and phytoalexins upon pathogen attack (González-Lamothe et al., 2009), inducing the expression of transporter-encoding genes in pathogens such as *B. cinerea* (Stefanato et al., 2009), *N. haematococca* (Coleman et al., 2011), and *M. oryzae* (Sun et al., 2007). It is also reported that fungal and oomycete ABC transporters also safeguard fungal cells against the intracellular accumulation of toxic biomolecules (Andrade et al., 2000) and facilitate the secretion of secondary metabolites (Patkar et al., 2012). A study on the impact of the fungicide metalaxyl on *P. infestans* reported that the induction of ABC transporters contributes to resistance against these chemicals by actively expelling the harmful substances from the parasite’s cells (Judelson and Senthil, 2006), possibly mediated by epigenetic mechanisms like chromatin modification (Childers et al., 2015; Leesutthiphonchai et al., 2018).

The transcript abundance of Phospholipase D (PLD) observed in PcPn_L was highly significant, up to 252-fold. Meijer et al. (2011) suggested that *Phytophthora* utilizes PLDs to alter host tissues, indicating a potential role for PLDs in pathogenicity. PLD functions by hydrolyzing structural phospholipids into their hydrophilic constituent and phosphatidic acid (PA). PA serves as the substrate for a highly conserved enzyme, phosphatidic acid phosphatase, which regulates lipid homeostasis by modulating cellular levels of both PA which is the substrate, and its product, diacylglycerol (Carman and Han, 2019). These lipids are crucial for synthesizing triacylglycerol and membrane phospholipids, as well as for various cellular processes such as lipid signaling, vesicular trafficking, lipid droplet formation, and the expression of genes involved in phospholipid synthesis (Carman and Henry, 2007; Carrasco and Mérida, 2007). Interestingly, in our study, the expression of phosphatidic acid phosphatase was found to be downregulated in PcPn_L, albeit to a lesser extent, while there was no significant change in expression observed in PcPn_E, compared to Pc. PA likely plays the role of a signaling molecule involved in vesicle formation and transport, cytoskeleton organization, protein transport, signal transduction, mitosis, etc. Previous studies have shown that the direct application of PA to plant leaves induces the expression of pathogenesis-related genes and leads to cell death (Meijer et al., 2019).

While model plants like *Nicotiana benthamiana* are frequently used for transient expression studies due to their ease of manipulation and high susceptibility to *Agrobacterium*, there are significant benefits to studying pathogen effectors in their natural host plants. Host-specific interactions between plants and pathogens can reveal unique aspects of the disease process that may not be apparent in model systems. These interactions include specific defense responses, signaling pathways, and gene expression profiles that are unique to the natural host. Varghese and Bhat (2011) developed a protocol for stable Agrobacterium-mediated transformation of black pepper plants via somatic embryogenesis which suggests that black pepper is not recalcitrant toward agrobacterium transformation. However, progress in developing genetic transformation technologies and subsequent gene-function assessments in black pepper has been limited since then. In the present study, we attempted a straightforward method for transient expression assays in black pepper leaves. A major difficulty encountered was that immersing black pepper leaves in GUS staining buffer led to tissue darkening, which obscured the characteristic blue coloration typically visible to the naked eye in other plants. To overcome this issue, we utilized microscopic examination, where the blue coloration was successfully observed, confirming the transient expression despite the initial visual obstacle. The apoplastic effector *PcNLP6* is a key contributor to the virulence of *P. capsici* in *C. annuum* (Feng et al., 2014). Another study in *N. benthamiana*, Park et al. (2023) successfully employed RNAi to suppress *P. capsici* infection by designing dsRNAs targeting *PcNLP6*. In our study, expression of *PcNLP6* is highly induced in PcPn_L. These factors collectively highlighted *PcNLP6* as a candidate of interest for studying its functional role in quick wilt disease of black pepper. Infiltration of *PcNLP6* resulted in a unique necrotic pattern in black pepper, which differed from the pattern observed in *N. benthamiana*. This discrepancy is likely due to variations in host and non-host responses to the effector. These findings underscore the need for further investigation to fully understand these responses.

## 5 Conclusion

*Phytophthora capsici* is a highly destructive pathogen responsible for significant losses in black pepper production. To gain insights into the molecular mechanisms underlying its pathogenicity, high-throughput sequencing was employed to track changes in *P. capsici* transcriptome during its interaction with black pepper. The study identified numerous potential pathogenicity candidates crucial for its infection in black pepper, highlighting targets for further research aimed at protecting this valuable spice crop. The study attempts to elucidate how the pathogen successfully colonizes its host. This includes deploying effectors to facilitate attack on the host’s innate immunity, defending against host counterattacks by expelling harmful compounds, and sustaining growth through flexible metabolic acquisition strategies. The findings unveil a captivating co-evolutionary molecular warfare between host and pathogen, opening new avenues for research on *P. capsici-*black pepper interactions. This study represents one of the first comprehensive investigations into *P. capsici* mycelial gene expression during infection of black pepper compared to its vegetative growth stage. Differential expression analysis highlights genes induced specifically for infection, shedding light not only on the role of effectors but also on changes in the transcription of transporters, cell wall-degrading enzymes, metabolic processes like glycolysis, and secondary messengers such as phosphatidic acid. These insights provide critical groundwork for advancing our understanding of *P. capsici* pathogenicity and for developing targeted strategies to mitigate quick wilt in black pepper.

## Data Availability

The datasets presented in this study can be found in online repositories. The names of the repository/repositories and accession number(s) can be found in the article/Supplementary material.

## Footnotes

^1^ https://phycocosm.jgi.doe.gov/Phyca11/Phyca11.home.html

^2^ mycocosm.jgi.doe.gov/Phyca11/Phyca11.home.html

^3^ www.bioinformatics.nl/cgi-bin/primer3plus/primer3plus.cgi

^4^ https://csbg.cnb.csic.es/BioinfoGP/venny.html
