# Supplementary material for: Transcriptomic analysis reveals pathogenicity mechanisms of Phytophthora capsici in black pepper
Source: Front Microbiol. 2024 Nov 18;15:1418816. doi: 10.3389/fmicb.2024.1418816 (PMC11609936; doi:10.3389/fmicb.2024.1418816)
Supplement: Supplementary file 1 [file Table_1.DOCX]

**Supplementary Material**

Supplementary Table 1. Primers used for RT-qPCR

| Name | Sequence | Amplicon size (bp) |
| --- | --- | --- |
| RxLR (e_gw1.225.17.1) | Forward: TTCTCCAAATATGGCGTTCC | 113 |
|  | Reverse: ATGCTTGGTTCCAGTCATCC |  |
| NLP (e_gw1.53.241.1) | Forward: AAGCCACAACTGGAAATTGG | 149 |
|  | Reverse: CATGTCGATCTGCCGTACAC |  |
| NLP (e_gw1.87.103.1) | Forward: GGGTGTCTACGCCTTCATGT | 118 |
|  | Reverse: GGGATTTTCCAGTGAGTCCA |  |
| NLP (e_gw1.547.4.1) | Forward: ACTGTCCCGACAAACTCTCG | 142 |
|  | Reverse: TCGGCTAATTCTGCTCGTTT |  |
| Elicitin (e_gw1.87.130.1) | Forward: TGGATCTGTCCGAGTGTGTG | 114 |
|  | Reverse: TGCTCGTAGTCGATCCAGTG |  |
| CRN (gw1.16.631.1) | Forward: GGGACGTTCCAAGAGTGTGT | 126 |
|  | Reverse: GAAGCCGTACAGGGAAAGTG |  |
