# Supplementary figures and images for: Transcriptomic analysis reveals pathogenicity mechanisms of Phytophthora capsici in black pepper
Source: Front Microbiol. 2024 Nov 18;15:1418816. doi: 10.3389/fmicb.2024.1418816 (PMC11609936; doi:10.3389/fmicb.2024.1418816)

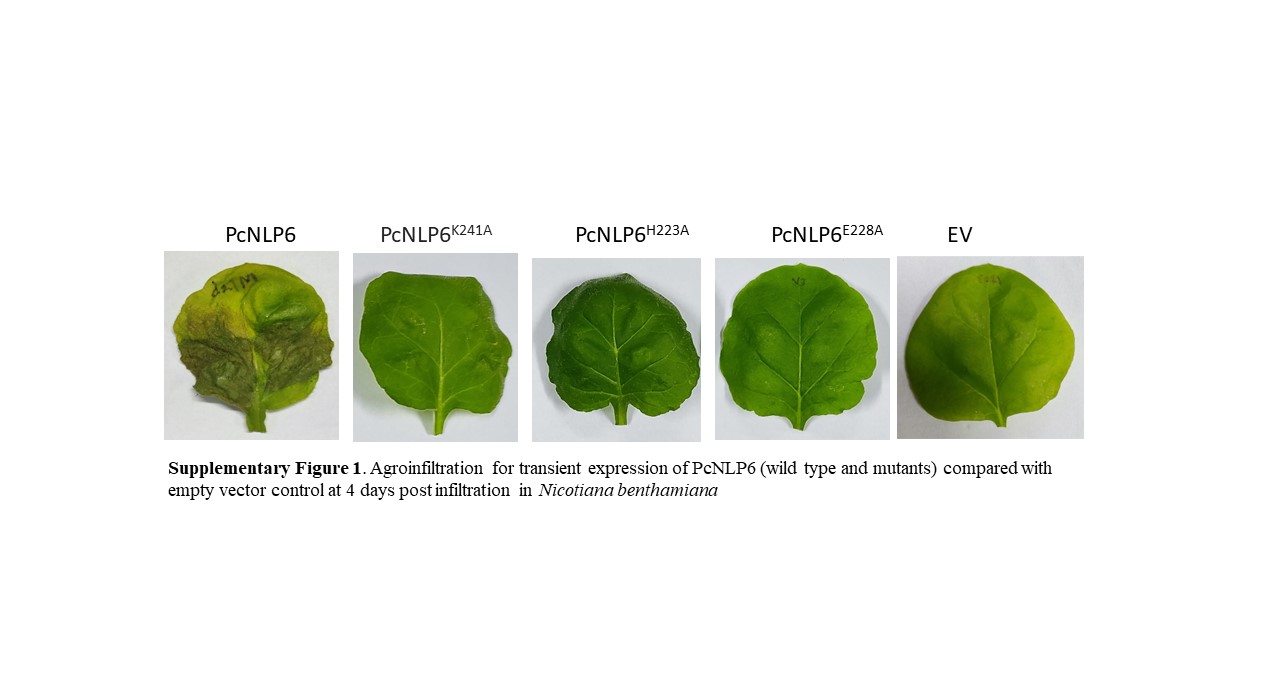

Supplement: Supplementary file 6 [file Image_1.JPEG]

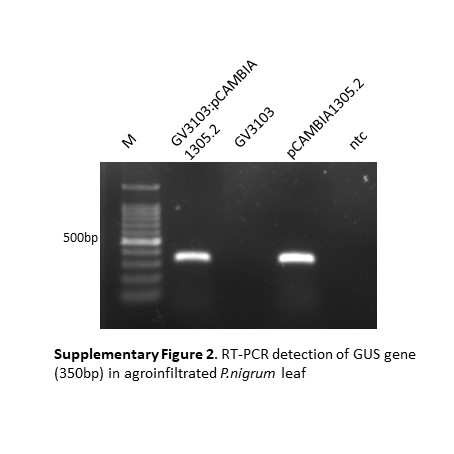

Supplement: Supplementary file 7 [file Image_2.JPEG]

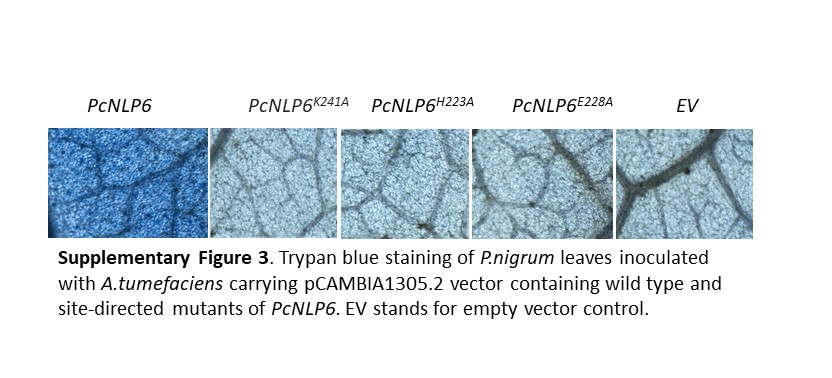

Supplement: Supplementary file 8 [file Image_3.JPEG]
